# Cell wall response of field grown *Populus* to *Septoria* infection

**DOI:** 10.3389/fpls.2023.1089011

**Published:** 2023-06-07

**Authors:** Nathan Bryant, Wellington Muchero, Rachel A. Weber, Jaime Barros, Jin-Gui Chen, Timothy J. Tschaplinski, Yunqiao Pu, Arthur J. Ragauskas

**Affiliations:** ^1^ Department of Chemical and Biomolecular Engineering, University of Tennessee, Knoxville, TN, United States; ^2^ BioEnergy Science Center & Center for Bioenergy Innovation, Oak Ridge National Laboratory, Oak Ridge, TN, United States; ^3^ Biosciences Division, Oak Ridge National Laboratory, Oak Ridge, TN, United States; ^4^ Division of Plant Sciences and Interdisciplinary Plant Group, University of Missouri, Columbia, MO, United States; ^5^ Department of Chemical and Biomolecular Engineering, University of Tennessee, Center for Renewable Carbon, Knoxville, TN, United States; ^6^ Department of Forestry, Wildlife, and Fisheries, University of Tennessee Institute of Agriculture, Knoxville, TN, United States

**Keywords:** *Septoria*, lignin, NMR, FTIR, PCA

## Abstract

Due to its ability to spread quickly and result in tree mortality, *Sphaerulina musiva (Septoria)* is one of the most severe diseases impacting *Populus*. Previous studies have identified that *Septoria* infection induces differential expression of phenylpropanoid biosynthesis genes. However, more extensive characterization of changes to lignin in response to *Septoria* infection is lacking. To study the changes of lignin due to *Septoria* infection, four field grown, naturally variant *Populus trichocarpa* exhibiting visible signs of *Septoria* infection were sampled at health, infected, and reaction zone regions for cell wall characterization. Fourier transform infrared spectroscopy (FTIR), nuclear magnetic resonance (NMR), and acid hydrolysis were applied to identify changes to the cell wall, and especially lignin. FTIR and subsequent principal component analysis revealed that infected and reaction zone regions were similar and could be distinguished from the non-infected (healthy) region. NMR results indicated the general trend that infected region had a higher syringyl:guaiacyl ratio and lower *p*-hydroxybenzoate content than the healthy regions from the same genotype. Finally, Klason lignin content in the infected and/or reaction zone regions was shown to be higher than healthy region, which is consistent with previous observations of periderm development and metabolite profiling. These results provide insights on the response of *Populus* wood characteristics to *Septoria* infection, especially between healthy and infected region within the same genotype.

## 1 Introduction

*Populus* has garnered interest as an economically important species for applications such as biofuel production due to its rapid growth, genetic diversity, and other advantageous attributes (Sannigrahi et al., 2010). One of the most critical drivers of biofuels’ economic feasibility is biomass yield (Chudy et al., 2019). Challenges to poplar growth productivity include susceptibility to fungal infections from pathogens such as *Sphaerulina musiva*, also known as *Septoria* (Feau et al., 2010). *Septoria* is particularly devastating due to its potential to be fatal to the tree and its ability to spread quickly among entire populations. Indeed, *Septoria* is considered one of the most severe diseases impacting hybrid poplar (Royle & Ostry, 1995). The two symptoms typically associated with *Septoria* infection are leaf spots and stem cankers (Cellerino, 1999). Leaf spots have a significant negative effect on photosynthesis and can lead to defoliation. Stem canker can cause broken tops, leading to severe growth penalties or death (Ostry and Mcnabb 1985; Ostry et al., 1989).

The secondary cell wall plays a role in many processes, including response to biotic and abiotic stress. It has been shown that lignin, a complex biopolymer that typically constitutes between 16 to 29% of *Populus* secondary cell walls (Bryant et al., 2020), is often synthesized and deposited at the site of fungal infections to form a periderm that prevents the spread of the pathogen. This result is consistent with previous studies of *Septoria* infection of *Populus*, with increased lignin deposition observed at the infection site. Previous transcriptome analyses of *Septoria* and other fungal infections have also identified differentially expressed genes in the phenylpropanoid and lignin biosynthesis pathway (Foster et al., 2015; Muchero et al., 2018). However, lignin content and/or structure are rarely reported in these types of studies. Additionally, these studies have evaluated response shortly after inoculation, and there is no information on the long-term effects of *Septoria* canker response in field-grown poplar. For instance, Bucciarelli et al. documented the differences in Klason lignin content and S/G ratio between susceptible and resistant *P. tremuloides* genotypes in response to *Entoleuca mammata* infection, but within 96 hrs of inoculation (Bucciarelli et al., 1998). To this effect, an exploratory study of four distinct field-grown *Populus* genotypes exhibiting signs of canker growth was conducted to determine if changes in lignin content and/or composition induced by *Septoria* infection could be elucidated. Healthy and infected region from each genotype was analyzed for Klason lignin content and composition by HSQC NMR. Additionally, whole-cell biomass was analyzed by FTIR.

## 2 Materials and methods

### 2.1 Sampling and preparation

Three-year-old *Populus trichocarpa* genotypes were sampled from a field site in Boardman, OR. The study was established in July 2016 using naturally varying genotypes from the *P. trichocarpa* genome-wide association mapping panel (Muchero et al., 2018). Samples of tree stems were taken at 20-30 cm above the soil line the form of approximately 2.5cm thick discs after 3 growing seasons in November 2018. Two hundred fifty-four out of 1,054 trees from this field site were observed to have stem and branch cankers in late summer 2018 – several months before the November 2018 harvest (Søndreli et al., 2020). Wood discs were oven-dried at 70°C for 14 days to remove moisture and prevent dry matter loss due to microbial activity. Five wood discs with noticeable signs of *Septoria* infection were selected for analysis. Two healthy discs were also selected for comparison. *Septoria* infection was previously verified at this field site using visual characterization as well as by isolation and sequencing of *S. musiva* isolates as described by Søndreli et al.(Søndreli et al., 2020). Briefly, wood from the margin between healthy and necrotic region was obtained from samples visually observed exhibiting stem canker. Region was plated on KV8 medium amended with streptomycin sulfate and chloramphenicol at 100 mg/L and 240 mg/L, respectively. The presence of *S. musiva* was confirmed by comparing the sequence of the ITS region (accession MN275180 to MN275187) to JX901814 with 99% identify. *S. musiva* infection results in a characteristic sunken stem canker that eventually leads to stem breakage. Diseased samples were selected on the basis of the severity of the infection which evident by discolorized wood.

Woody biomass for analysis was obtained by drilling the discs with a 13mm spade drill bit and collecting the wood shavings. Each disc was drilled at three locations: (1)in the healthy region of the sapwood to capture xylem with no signs of infection; (2) in the discolored *Septoria* infected region; and (3)along the reaction zone between the infected region and healthy xylem, herein referred to as the reaction zone. Each drilling location was selected to be a consistent distance from the pith to avoid the effects of radial variation. Drilling for sample material was done between the pith and the bark. The drill was cleaned between each use to mitigate cross-contamination. Each sample was Wiley-milled using a 40-mesh screen, and the mill was cleaned between each use. Milled wood samples were Soxhlet extracted with toluene/ethanol (2:1, v:v) overnight to remove extractives and then air-dried in a fume hood for at least 48 h. Extractives-free biomass was used in all further analyses.

### 2.2 Fourier-transform infrared spectroscopy

Extracted biomass was analyzed by FTIR *via* a Perkin Elmer Spectrum 100 FTIR spectrometer with a universal attenuated total reflection (ATR) accessory and collected *via* Spectrum software. A background scan was performed prior to sample analysis. Extracted samples were analyzed from 4,000-600 cm^-1^ with a resolution of 2 cm^-1^ and 32-scan accumulation. Each sample was analyzed in triplicate, and the three spectra were averaged for further analysis. The resulting spectra were subsequently processed and analyzed with OriginPro software. All spectra were baseline corrected manually using twelve anchor points and normalized around the 1505 cm^-1^ peak. PCA modeling was performed within the OriginPro software environment using spectral data in the fingerprint region of 1800-600 cm^-1^.

### 2.3 Heteronuclear single quantum coherence nuclear magnetic resonance spectroscopy

To prepare samples for HSQC analysis, extracted biomass was ball milled at 600 RPM for 2 hours to obtain a fine powder. Each sample was then combined with cellulase (Cellulysin) in an acetate buffer (pH 5.0) and shaken on an incubator shake at 35°C for 48h. After centrifugation, the supernatant was discarded, and the solid residue was recovered and washed with DI water three times. The solid residue was then lyophilized for at least 48h to produce enzyme lignin (EL). EL was dissolved in DMSO-d_6_ in a 5mm NMR tube, sonicated for 1h, and allowed to swell overnight before analysis. For whole cell wall (WCW) analysis of BESC-335, powdered material was transferred to a 5mm NMR tube for direct dissolution with DMSO-d_6_/HMPA-d_18_ (4:1). All NMR spectral data were recorded using a Bruker Avance III HD 500 MHz spectrometer. A standard hsqcetgpsip2.2 Bruker pulse sequence was used with an N_2_ cryoprobe with the following specifications: ^1^H spectra width of 12 ppm and 1024 data points; ^13^C spectra width of 220 ppm with 256 increments and 32 scans. All HSQC spectra were analyzed with Bruker TopSpin 3.5pl6 software. The DMSO-d_6_ solvent peak at δ_C_/δ_H_ 39.5/2.49 was used to calibrate the spectra.

### 2.4 Klason lignin analysis

The Klason lignin content of each sample was determined based on modified NREL established procedures (Sluiter et al., 2008). Briefly, extracted biomass was first dried overnight at 45C in a vacuum oven. The first hydrolysis was performed with 78% sulfuric in a 30°C block heater for 1 hour with mixing every 5-10 minutes. For the second hydrolysis, the mixture was diluted to 4% sulfuric acid by adding DI water and then autoclaved at 121°C for 1 hour. The acid insoluble residue (AIR) and filtrate were separated by vacuum filtration. The AIR was dried at 105°C overnight and weighed for Klason lignin determination.

### 2.5 Lignin composition analysis by GC/MS

Thioacidolysis was performed on EL residues that were recovered from HSQC NMR analysis. The EL residues recovered from the NMR tube by precipitation through the addition of DI water, centrifugation, and decanting the DMSO-d_6_/DIW supernatant. The recovered EL was then lyophilized for 48h. Thioacidolysis was then performed to determine the lignin monomer composition using gas chromatography mass spectrometry (GC/MS), as previously described (Barros et al., 2019; Chen et al., 2021). Briefly, 12 ml of thioacidolysis reagent was made containing 10.5 ml of 1,4-dioxane, 0.3 ml of boron trifluoride diethyl etherate, and 1.2 ml of ethanethiol. Bisphenol E in 1,4-dioxane was added as internal standard with final concentration of 1.42 mg/ml. The reagent was vortexed, and 500 µl were added to 3 mg of lyophilized EL residues. Samples were then incubated at 100°C for 2 hours and vortexed every 45 minutes. After incubation, samples were cooled to room temperature. A total of 250 µl of the supernatants were transferred to 4 ml vials and 95 µl of saturated NaHCO_3_ was added. Samples were dried down at 40°C under a slow flow of nitrogen gas. For derivatization, a pyridine:BSTFA (1:1) solution was made, and 100 µl were added to each sample. Samples were then incubated in an orbital shaker for 30 minutes at 37°C with gentle agitation. Finally, 50 µl of each sample were transferred to GC vials and sent for GC-MS analysis. GC/MS was performed on an Agilent 6890N GC with a 5973N series MS detector with a DB-5 ms capillary column (60 m × 0.25 mm × 0.25 μm film thickness). Mass spectra were recorded in electron impact mode (70 eV) with 60–650 m/z scanning range. The ions extracted were 239, 269, 299, and 347 m/z for the thioethylated coumaryl (H), coniferyl (G), and syringyl (S) monomers, and internal standard bisphenol E, respectively.

### 2.6 Chemical analysis of *p*-hydroxybenzoate

The amount of *p*-hydroxybenzoate (PB) was determined by an established method (Goacher et al., 2021) with slight modifications. Briefly, 1mL of 2M sodium hydroxide and 100μL of 1mg/mL *o*-coumaric acid (internal standard) was added to 20mg of extractive-free powder. Samples were incubated at 30°C for 24h and the reaction was subsequently terminated by the addition of 100μL of 72% sulfuric acid. Samples were then incubated on ice for 5 minutes. The supernatant was collected by centrifugation and filtered through a 0.45μm nylon syringe filter prior to high-performance liquid chromatography (HPLC) analysis.

Samples were analyzed by an Agilent 1200 series HPLC. For each sample, 10μL was injected onto a Symmetry C18 column (4.6 x 50mm, 5μm particle size) maintained at 35°C. Adequate peak resolution was achieved using 75:25 (v:v) ratio of eluent B (0.1% trifluoroacetic acid in 70:30 acetonitrile:methanol) in eluent A (0.1% triflouroacetic acid in water) at a flow rate of 0.4mL/min. Spectra were integrated at the UV maxima of 255nm and a five-point calibration curve was used for quantification. The PB measurement obtained by HPLC was normalized by lignin content as determined by the Klason lignin method for comparison to HSQC NMR results.

## 3 Results

**Figure 1:**
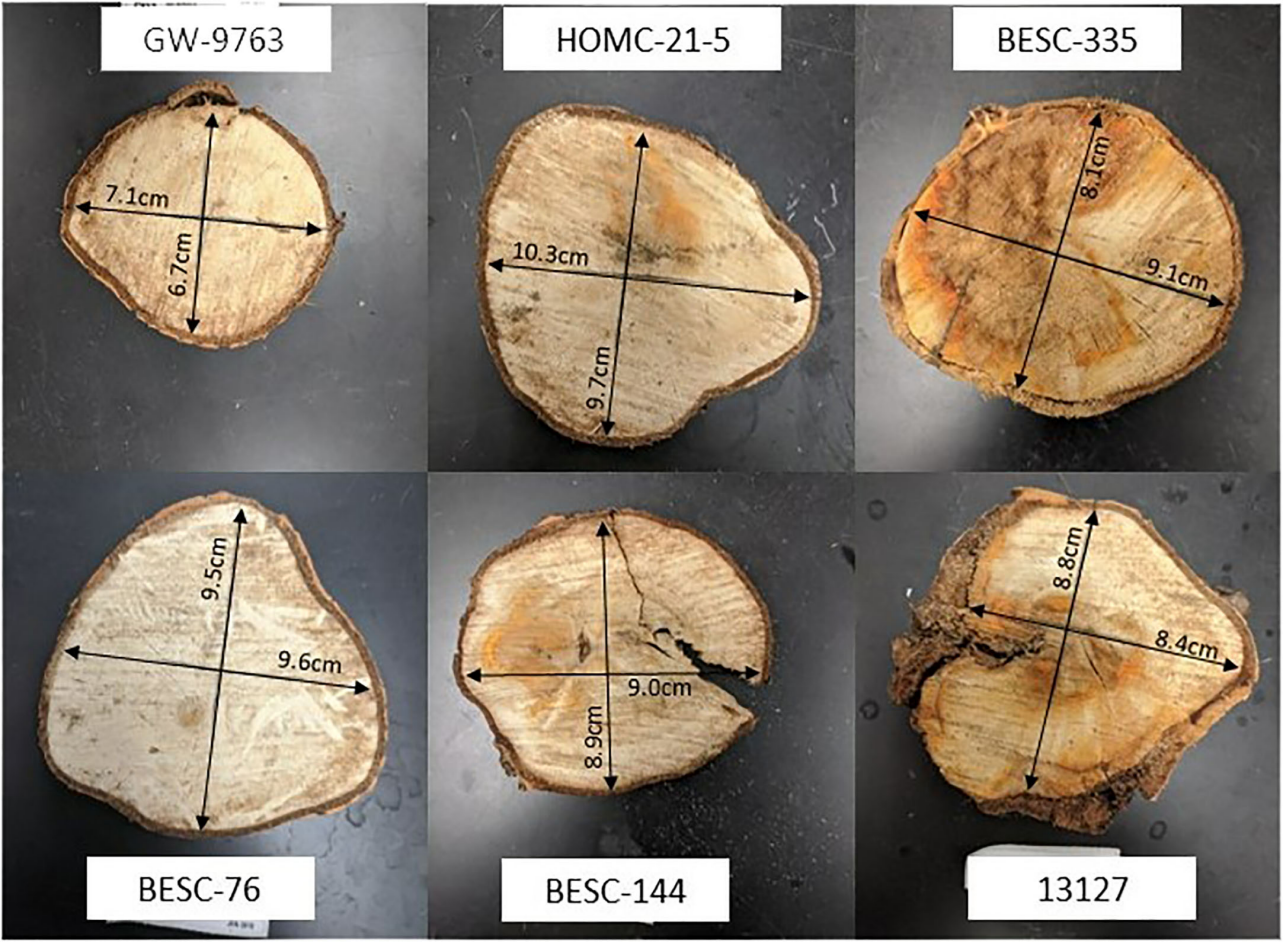
Wood discs from six lines were selected for analysis. The wood discs from four lines exhibiting signs of *Septoria* infection are 13127 (bottom right), HOMC-21-5 (top middle), BESC-144 (bottom middle), and BESC-335 (top right). Wood discs from lines GW-9763 (top left) and BESC-76 (bottom left), exhibiting no signs of *Septoria* infection, were utilized as controls.

Careful consideration was taken when selecting the sampling locations on each wood disc. One specific concern was radial variation. It is well documented that wood structure exhibits radial variation between the pith and the bark (Lachenbruch et al., 2011). Therefore, each sample was collected at a consistent radius (between approximately 7.6cm and 10.2cm) from the center of the pith to mitigate radial differences. A total of four *Septoria* infected discs were selected for analysis, as displayed in **Figure 1**. Of these, two genotypes (13127 and BESC-335) exhibited more severe signs of infection, covering an estimated 50-75% of the observable surface area. The other two samples (HOMC-21-5 andBESC-144) exhibited less severe signs of infection, with spots covering an estimated 25% or less of the surface area.

### 3.1 FTIR and PCA

**Figure 2:**
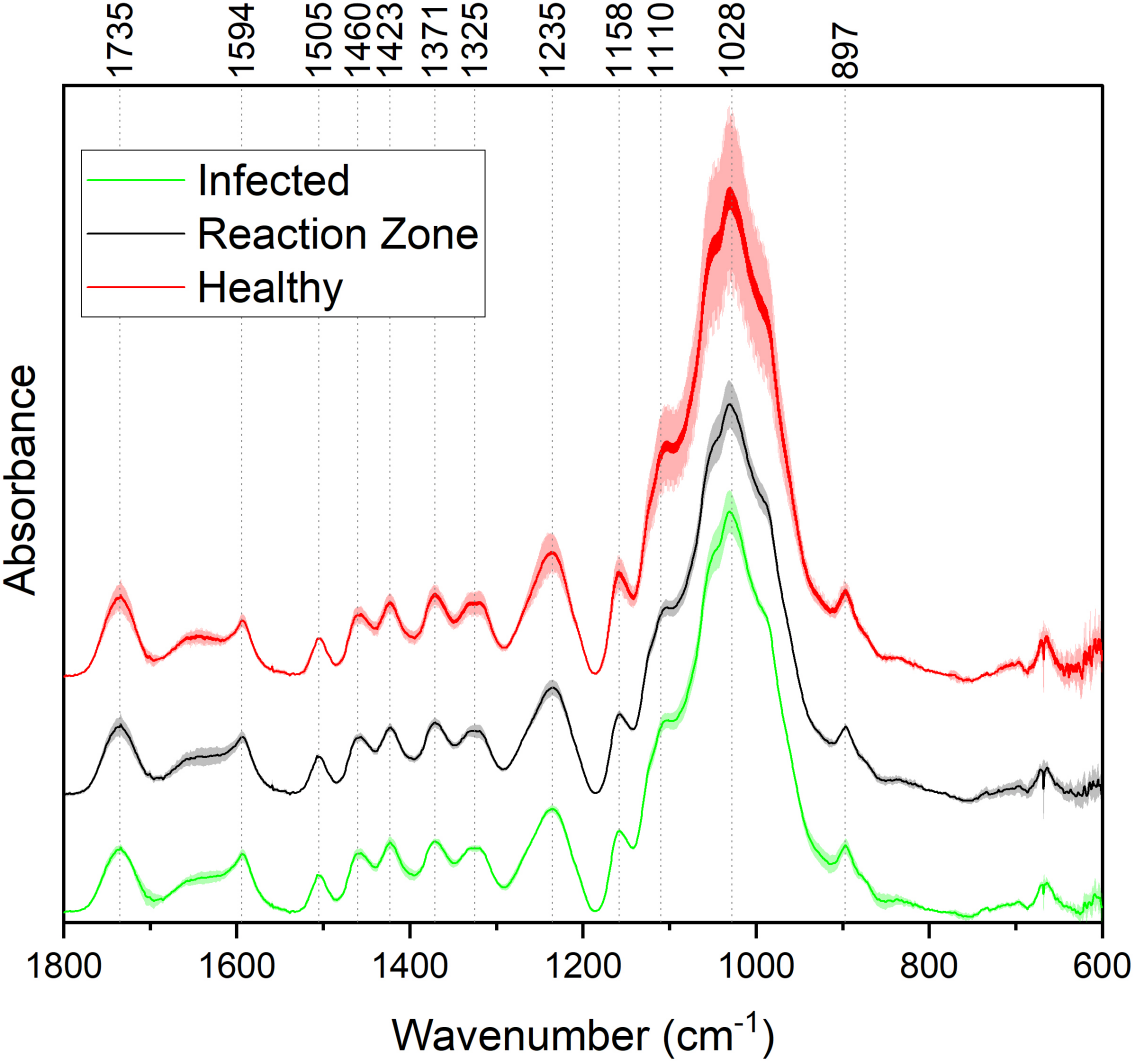
FTIR absorbance spectra of healthy (red), reaction zone (black), and infected (green) extracted biomass. For each spectrum, each of the different regions (healthy, reaction zone, infected) was pooled across the four genotypes. For each region across the four genotypes, the average values (N=4) are plotted as a solid line and the standard deviation is represented by the associated shaded area.

FTIR analysis of extracted biomass was measured in the range of 4000-600 cm^-1^. All samples were run in triplicate, and the three spectra were averaged to account for variability. The averaged spectra were baseline corrected and normalized around the 1505 cm^-1^ peak. **Figure 2** displays spectra, pooled by region, for the fingerprint region of 1800-600 cm^-^. The average values are represented by the solid lines, with the standard deviations represented by the surrounded bands of the same color. Peak assignments were made according to the literature and are summarized in **Table 1** (Xu et al., 2005; Kline et al., 2010; Zhou et al., 2011; Akinosho et al., 2017; Shi et al., 2019; Yang et al., 2020). Several peaks typically associated with biomass and lignin are observed in this region. Notably, the large band at 1028 cm^-1^ is associated with C-O stretching vibrations from primary alcohols. Another band associated with the C=O stretching in hemicellulose (xylan) was also observed at 1735 cm^-1^. Peaks around 1158 cm^-1^ and 897 cm^-1^ were observed, corresponding to the C-O-C vibrations of ester groups and C-H deformations of cellulose, respectively. Bands characteristic of the aromatic skeletal vibrations of lignin at 1505 cm^-1^ and 1594 cm^-1^ were also observed. Additionally, a peak at 1325 cm^-1^, associated with syringyl ring breathing with a CO stretching, was observed in all samples. Changes in the peak intensity between healthy, reaction zone, and infected regions can provide insight to the effects of fungal degradation. As summarized in **Table 2**, there were several changes in the intensity ratios between the various regions. Compared to the healthy region, the reaction zone and infected regions exhibited a general increase in the 1594cm^-1^ intensity, indicating an increase in lignin content. There were also decreases in the 1157cm^-1^ peak from the healthy to reaction zone and infected regions, suggesting differences in *p*-hydroxybenzoate content. Both the 1325cm^-1^ and 1235cm^-1^ peaks also exhibited changes in intensities between the various regions. The ratio of these two peaks was correlated with the NMR S/G ratio (CC=0.54), indicating changes in the S/G ratio consistent with NMR measurements.

**Table 1:** Assignment of peaks in the FTIR spectra.

Observed Peak (cm^-1^)	Peak Assignment	Reference
1735	C=O stretching in lignin and hemicellulose	(Yang et al., 2020)
1594	Aromatic skeletal vibrations and C=O stretching in lignin	(Xu et al., 2005; Zhou et al., 2011; Yang et al., 2020)
1505	Aromatic C=C skeletal vibrations in lignin	(Xu et al., 2005; Zhou et al., 2011; Shi et al., 2019; Yang et al., 2020)
1460	C-H bending of methyl and methylene groups	(Kline et al., 2010; Zhou et al., 2011; Shi et al., 2019; Yang et al., 2020)
1423	C-H deformation, CH_2_ bending vibration, carboxyl group stretching	(Xu et al., 2005; Kline et al., 2010; Zhou et al., 2011; Yang et al., 2020)
1371	C-H bending, stretching	(Shi et al., 2019)
1325	C=O stretching of syringyl units	(Kline et al., 2010; Zhou et al., 2011; Akinosho et al., 2017; Shi et al., 2019; Yang et al., 2020)
1235	C-C, C-O, and C=O stretching of guaiacyl unit	(Zhou et al., 2011; Akinosho et al., 2017; Yang et al., 2020)
1158	C-O stretching of ester group	(Xu et al., 2005; Kline et al., 2010; Shi et al., 2019)
1110	Aromatic C-H deformation of syringyl units	(Kline et al., 2010; Yang et al., 2020)
1028	C-O stretching of primary alcohols	(Kline et al., 2010; Shi et al., 2019; Yang et al., 2020)
897	C-H deformation vibration of cellulose	(Kline et al., 2010)

**Table 2:** The ratios of peak intensities between healthy and reaction zone/infected regions are compared to indicate changes to the cell wall upon pathogen infection.

Genotype	Region	Wavelength Ratio Compared to Healthy							
		1735 cm^-1^	1594 cm^-1^	1370 cm^-1^	1325 cm^-1^	1235 cm^-1^	1157 cm^-1^	1120 cm^-1^	897 cm^-1^
13127	Reaction Zone	0.95	1.06	0.98	1.00	0.98	0.93	0.96	0.97
	Septoria	1.05	1.12	1.04	1.05	1.06	0.98	1.07	1.01
HOMC-21-5	Reaction Zone	1.02	1.00	0.87	0.83	0.97	0.80	0.88	0.79
	Septoria	0.89	0.98	0.84	0.85	0.86	0.78	0.81	0.84
BESC-144	Reaction Zone	0.72	1.08	0.77	0.77	0.77	0.71	0.73	0.69
	Septoria	0.74	1.17	0.76	0.82	0.79	0.72	0.81	0.58
BESC-335	Reaction Zone	0.83	1.03	0.90	0.89	0.76	0.70	0.74	0.74
	Septoria	0.66	1.00	0.87	0.83	0.71	0.73	0.74	0.76

The spectral information from the fingerprint region of 1800-600 cm^-1^ was considered for the PCA. The resulting PC1 and PC2 explained 92.6% and 3.4% of the variation, respectively. Subsequent PCs accounted for less than 2% of the variation each. PC loadings were examined to determine which bands contributed to each principal component. For PC1, the band around 1028cm^-1^ was determined to be most significant contributor. This band has been associated with various properties of biomass and lignin, such as the C-O-C bonds in β-O-4 aryl ether linkages and other C-O bonds linked to primary and secondary alcohols. The peak around 1110 cm^-1^, associated with the C-H deformation of S units, also contributed to PC1. Loading analysis for PC2 revealed that bands around 1640 cm^-1^ and 1735 cm^-1^ contributed most significantly to this principal component. These bands are associated with C=O stretching in lignin and xylan, respectively. The PCA output including eigenvalues, loadings, and scores are provided in the **Supplementary Material**.

### 3.2 HSQC NMR

**Figure 3:**
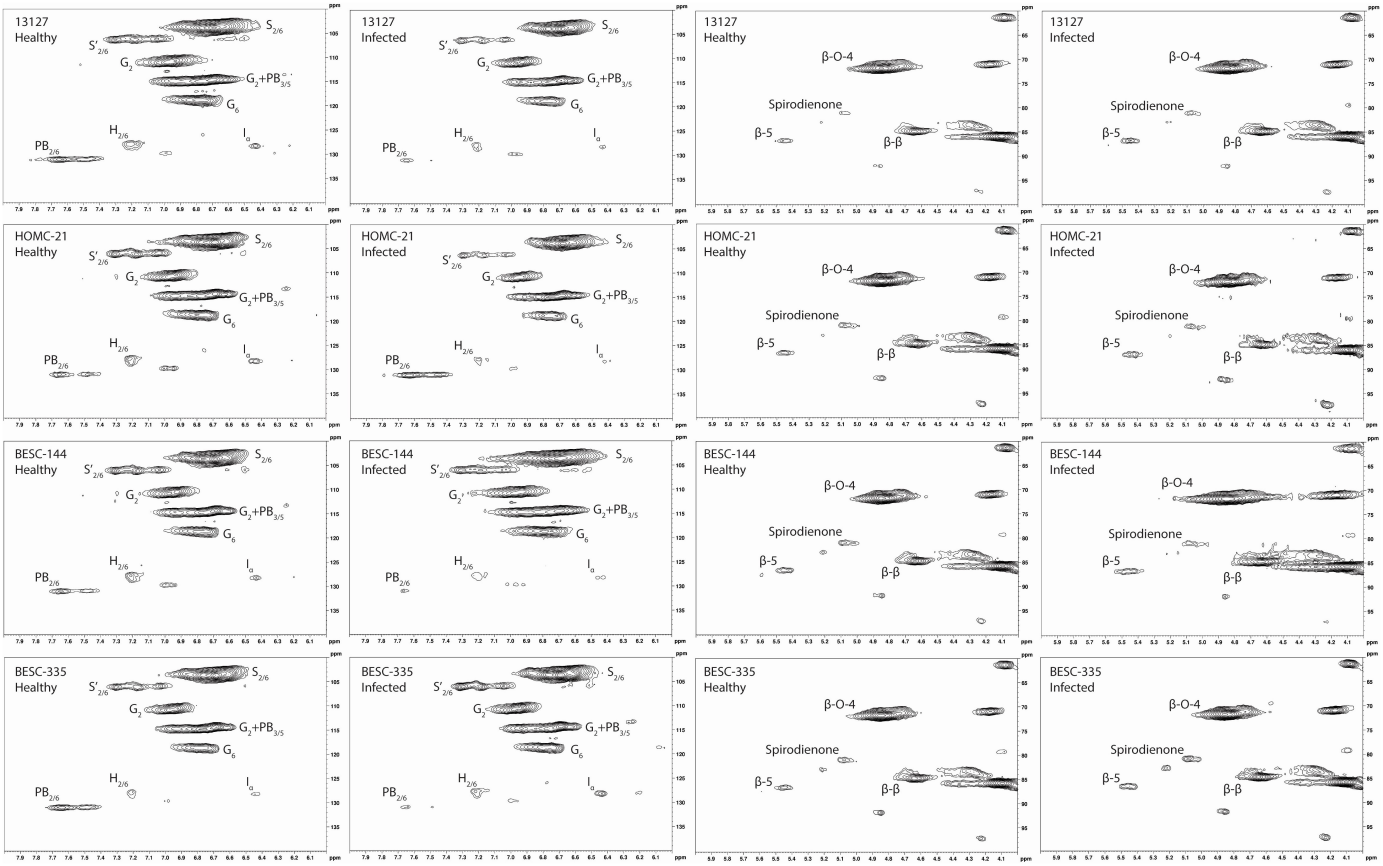
Aromatic and aliphatic region plots of the HSQC NMR spectra of the isolated lignin from each of the four analyzed lines. The first column contains the aromatic region of the healthy region from each sample. The second column contains the aromatic region of the infected region. The third column contains the aliphatic region of the healthy region. The fourth column contains the aliphatic region of the infected region. The HSQC NMR spectra of the healthy control sample GW-9763 is available in the supplementary material (**Figure S3**) for comparison.

Each *Populus* sample was subjected to enzymatic hydrolysis, and 2D HSQC NMR was employed to analyze the resulting enzyme lignin fraction to examine lignin structure and identify differences between healthy and infected region. The aromatic region of the HSQC spectra for the healthy and infected region for each line is shown in **Figure 3**.

All spectra indicated the presence of the expected aromatic syringyl (S), guaiacyl (G), and p-hydroxyphenyl (H) units, as observed in **Figure 3**. The S units exhibited a strong S_2,6_ correlation at δ_C_/δ_H_ 103.8/6.70 ppm. Correlations associated with G units were observed at δ_C_/δ_H_ 111.0/6.98 ppm (G_2_), 115.1/6.72, 6.98 ppm (G_5_), and 119.1/6.80 ppm (G_6_). The H_2/6_ peak was also observed at 128.2/7.17 ppm. Other correlations observed in the aromatic region include *p*-hydroxybenzoate (PB_2/6_) at δ_C_/δ_H_ 130.4/7.62 ppm and cinnamyl alcohol (I_α_) at δ_C_/δ_H_ 128.3/6.45 ppm. All spectra also exhibited peaks in the aliphatic region associated with β-O-4, β-5, β-β, and spirodienone interunit linkages. Relative abundance of each lignin structural feature is summarized in **Table 3**. Samples were independently analyzed by thioacidolysis for lignin composition. The ratio of S units to G units (S/G ratio) determined by thioacidolysis and HSQC NMR are highly correlated (Pearson correlated coefficient = 0.63; *p*-value < 0.01), thereby validating the S/G ratio measurements. The relative content of H units as determined by thioacidolysis and NMR were also compared. The H unit content exhibited poor correlation between the two methods (Pearson correlation coefficient = -0.1; *p*-value = 0.7). It has previously been shown that the HSQC NMR signal for H_2/6_ can be overlapped by various proteins, leading to overestimation of H unit content (Kim et al., 2017). We expect this to be the case here. Therefore, careful consideration was given to results depending on the interpretation of H unit content. Thioacidolysis results are provided in the **Supplementary Material**.

**Table 3:** Semi-quantitative results from integration of the HSQC NMR spectra.

Sample	Status	S	G	H	PB	S/G	β-O-4	β-5	β-β	Spirodienone
13127	Healthy	67.4	31.5	1.11	4.80	2.14	58.8	3.56	7.57	1.30
	Reaction Zone	67.7	31.3	1.03	2.01	2.16	60.1	3.19	8.51	1.34
	Infected	68.2	29.9	1.95	2.96	2.28	57.9	3.18	8.14	1.37
HOMC-21-5	Healthy	70.4	28.1	1.52	3.69	2.50	60.4	2.73	7.46	1.64
	Reaction Zone	69.7	29.6	1.72	2.35	2.35	62.7	2.15	7.78	0.81
	Infected	69.1	29.1	1.81	8.22	2.38	57.2	2.53	5.70	0.85
BESC-144	Healthy	68.5	29.0	2.55	4.26	2.37	56.9	3.02	7.52	1.66
	Reaction Zone	67.7	30.0	2.36	3.62	2.26	64.7	3.18	8.11	1.56
	Infected	69.4	29.4	1.18	1.98	2.36	61.3	2.87	8.08	1.28
BESC-335	Healthy	70.5	27.6	1.84	6.42	2.55	58.5	3.02	8.20	1.57
	Reaction Zone	71.1	27.6	1.35	3.94	2.58	58.0	2.48	7.81	1.47
	Infected	71.4	26.6	1.93	1.74	2.68	58.0	2.75	8.49	1.63

Signals used for volume integration are as follows: δ_C_/δ_H_ 103.8/6.70 ppm for S_2/6_, δ_C_/δ_H_ 111.0/6.98 ppm for G_2_, δ_C_/δ_H_ 128.2/7.17 ppm for H_2/6_, δ_C_/δ_H_ 130.4/7.62 ppm for PB_2/6_, δ_C_/δ_H_ 128.3/6.45 ppm for I_α_, δ_C_/δ_H_ 71.8/4.86 for β-O-4, δ_C_/δ_H_ 86.8/5.46 for β-5, δ_C_/δ_H_ 84.8/4.65 for β-β, and δ_C_/δ_H_ 81.2/5.07 for spirodienone. Results are presented relative to an S+G+H basis.

Similarly, NMR may overestimate PB since it exists as a terminal unit. Therefore, the PB content of a subset of samples was determined *via* alkaline hydrolysis and HPLC. Due to material availability, not all samples were analyzed. However, the two samples with the highest PB content as measured by NMR (HOMC-21-5 infected, 8.22%; BESC-335 healthy, 6.42%) were included for this analysis. The PB content of each sample was normalized by its associated Klason lignin content. While HPLC measured a lower absolute value of PB (0.73%-1.44%) than NMR (1.61%-8.22%), the two measurements were highly correlated (R^2 = ^0.71; *p*-value=0.002). The alkaline hydrolysis method also identified samples HOMC-21-5 infected and BESC-335 healthy as having elevated PB content (1.44% and 1.22%, respectively). To further validate this measurement, a sample that has previously been utilized as an internal reference (denoted as BESC standard poplar (Bhagia et al., 2016)), was also included in this analysis. This sample was run in duplicate *via* HPLC and in triplicate *via* HSQC NMR. The PB content of this sample was measured to be 2.13% ± 0.20% by HPLC and 16.27% ± 0.36% by HSQC NMR. This point fits the trendline established by the ten samples from this study that were analyzed, providing greater confidence of the correlation between the two measurements. Additional information regarding this comparison can be found in thesupplemental material (**Figure S3**, **Table S2**).

To explore potential changes in cell wall polysaccharides, the healthy, reaction zone, and infected regions of sample BESC-335 were subjected to whole cell wall (WCW) HSQC NMR by directly dissolving extractive free, ball milled biomass in a DMSO-d_6_/HMPA-d_18_ (4:1) solvent system. Resulting spectra are included as a figure in the supplemental material (**Figure S6**) In the non-anomeric region (δ_C_/δ_H_ 50-90/2-6) several cellulose signals were observed, including internal cellulose units (CI_4_, CI_5_, CI_6_). Additionally, the non-reducing ends (CNR_3_, CNR_5_) of cellulose were identified. The non-anomeric region also contained many xylan related signals, such as xylan internal units (XI_2_, XI_4_, XI_5_), reducing ends (XRα_4_, XRβ_4_), and non-reducing ends (XNR_2_). Hardwoods contain acylated xylan, and associated acetylated xylan signals (2-*O*-Ac-β-D -Xyl, 3-*O*-Ac-β-D-Xyl) were also observed. In the anomeric (δ_C_/δ_H_ 90-105/3.5-6) region, the signal for internal cellulose [(1→4)-β-D-Glc*p*] was observed, though the signal for the cellulose non-reducing end [(1→4)-β-D-Glc*p* (NR)] was generally better resolved. The signal associated with internal xylan units [(1→4)-β-D-Xyl*p*] was also prominent. 4-O-methyl-α-D-glucuronic acid (4-O-MeGlcA) was readily observed in the reaction zone and infected regions. This signal was present in the healthy region, though at levels very close to background. In summary, while some differences between the different regions were observed, the healthy, reaction zone, and infected regions appear to largely contain similar cellulose and hemicellulose structures.

### 3.3 Klason lignin analysis

**Figure 4:**
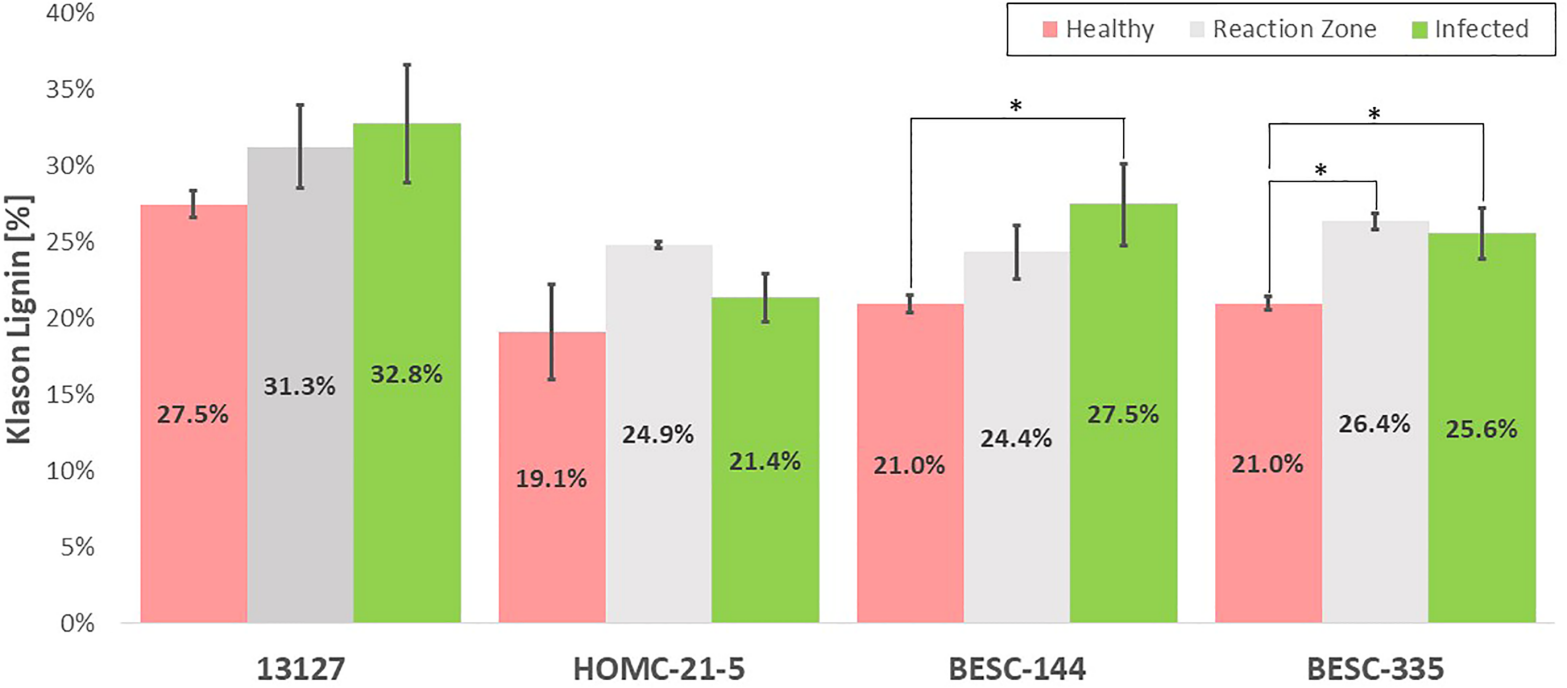
Klason lignin content of the healthy (red), reaction zone (gray), and infected (green) region of each genotype as determined by two-step acid hydrolysis. One stars (*) associated with reaction zone or infected regions indicate a statistically significant difference (*p*-value < 0.05) compared to the health region of the same genotype. A comparison with healthy control sample GW-9763 is available in the supplementary material (**Figure S4**) for comparison.

The Klason lignin content the health, reaction zone, and infected regions of each sample was determined gravimetrically after two-step acid hydrolysis (Sluiter et al., 2008). As depicted in **Figure 4**, the lignin content was generally elevated in the reaction zone and/or infected regions compared to the healthy region. Both genotypes 13127 and BESC-144 exhibited consistent increases in lignin content from the healthy to infected regions. The lignin content of genotype 13127 increased from 27.5% ( ± 0.9%) in the healthy region to 31.3% ( ± 2.7%) and 32.8% ( ± 3.9%) in the reaction zone and infected regions, respectively. Similarly, the lignin content of genotype BESC-144 was measured to be 21.0% ( ± 0.6%) in the healthy region, 24.4% ( ± 1.8%) in the reaction zone region, and 27.5% ( ± 2.7%) in the infected region. BESC-335 also exhibited higher lignin content around the infection site (25.6% ± 1.7%) and reaction zone region (26.4% ± 0.5%) than the healthy region, which measured 21.0% ( ± 0.4%) lignin. While the infected region HOMC-21-5 exhibited lignin content (21.4% ± 1.6%) similar to the healthy region (19.1% ± 3.1%), the associated reaction zone region was measured to have a much higher lignin content of 24.9% ( ± 0.2%).

## 4 Discussion

### 4.1 FTIR and PCA

FTIR has been widely utilized as a non-destructive method of rapidly analyzing biomass properties, and the assignments of spectra peaks are well documented. Perhaps the most significant finding is the apparent S/G ratio agreement between FTIR and NMR. The ratio of the 1325cm^-1^ and 1235cm^-1^ peaks from the FTIR spectra has previously been used as a predictor for the S/G ratio (Kline et al., 2010). The ratio of these peaks across all samples were found to be correlated with the NMR S/G ratio (**Table 4**), supporting the relative S/G ratio measurements.

**Table 4:** The S/G ratio measured by FTIR, NMR, and HSQC NMR are summarized.

Genotype	Region	S/G ratio		
		FTIR	NMR	Thioacidolysis
13127	Healthy	0.58	2.14	1.91
	Reaction Zone	0.59	2.16	1.99
	Septoria	0.57	2.28	1.82
HOMC-21-5	Healthy	0.63	2.50	1.97
	Reaction Zone	0.53	2.35	2.03
	Septoria	0.62	2.38	1.93
BESC-144	Healthy	0.63	2.37	2.01
	Reaction Zone	0.63	2.26	1.84
	Septoria	0.66	2.36	1.83
BESC-335	Healthy	0.58	2.55	–
	Reaction Zone	0.67	2.58	1.99
	Septoria	0.68	2.68	2.15

The S/G ratio measured by HSQC NMR are correlated with FTIR and thioacidolysis as indicated by associated Pearson correlation coefficients of 0.54 and 0.62, respectively.

While FTIR is a convenient analytical technique, trends and differences between samples can often be difficult to elucidate. Therefore, principal component analysis (PCA) was utilized to extract additional information from the spectra. PCA is a valuable dimension reduction technique that emphasizes differences in samples based on spectral variation and has been used extensively for analyzing biomass and lignin (Xu et al., 2013). The resulting score plot (available in the supplemental material as **Figure S7**) indicates that reaction zone (black) and infected (green) regions are quite similar. Additionally, PCA does a reasonable job at distinguishing the healthy region (red) from the reaction zone and infected regions, even though the healthy region of HOMC-21-5 falls just inside the 95% confidence interval (CI) of the reaction zone and infected regions. The exception is sample 13127, where the PCA identifies the healthy region to be more characteristically similar to reaction zone and infected region samples. These results suggest that sample 13127 may exhibit a cell wall structure similar to *Septoria* infection that are not immediately visible. This is supported by the Klason lignin measurements, as the healthy region of 13127 has significantly higher lignin content than the healthy region from HOMC-21-5, BESC-144, and BESC-335 (**Figure 4**). These FTIR and PCA results confirm that there are clear changes to cell wall structure induced by *Septoria* infection.

### 4.2 HSQC NMR

Changes in the S/G were observed between the healthy and infected region of the more severely infected samples. Genotype 13127 exhibited differences in S/G ratios, measuring 2.14 in the healthy region but increased to 2.43 in the infected region. The S/G ratio also increased across region condition in genotype BESC-335, measuring 2.55, 2.58, and 2.68 in the healthy, reaction zone, and infected regions, respectively. This would be consistent with the observed upregulation caffeate O-methyltransferase (COMT) due to *Septoria* infection (Foster et al., 2015). COMT is involved in the methylation of monolignols, and its upregulation would increase the ratio of S lignin (Do et al., 2007). However, the samples exhibiting lower severity of infection did not exhibit this trend. For the HOMC-21-5 genotype, the S/G ratio was higher in the reaction zone region (2.83) than the healthy (2.50) or infected (2.38) regions. The second low severity genotype BESC-144, exhibited a similar S/G ratio in the healthy (2.37) and infected (2.36) region, but a decreased S/G ratio (2.26) in the reaction zone region. All samples exhibited relatively low and consistent amounts of spirodienone (1.38 ± 0.26) and β-5 (2.89 ± 0.38) linkages. As expected, the most abundant interunit linkage in all samples was the β-O-4 aryl ether linkage. The variation of β-O-4 abundance was slightly more variable (σ=3.01) in the low severity genotypes (HOMC-21-5, BESC-144) than in the high severity (σ=0.86) genotypes (13127, BESC-335). The β-β and spirodienone linkages exhibited a similar trend of higher variability in the low severity genotypes. It has historically been demonstrated that β-O-4 content exhibits a strong positive correlation to the S/G ratio (Yoo et al., 2018), due in part to the propensity of S units to form this linkage. However, the four *Septoria* infected genotypes samples did not conform to this trend. Additionally, the S/G ratio was not correlated with either the β-β or spirodienone linkages. This is surprising, as both of these linkages have been shown to correlate with syringl unit content (Stewart et al., 2009). The one trend that was observed across these twelve samples was the negative correlation between the S/G ratio on the β-5 content (*p*-value < 0.001). This correlation is expected, as G lignin can undergo coupling at the 5-position. A lack of correlation between the S/G ratio and all linkage types was observed in the control samples. This could be contributed to relatively low abundance and limited variation in some of the linkage types. For instance, across the twelve samples taken from the infected wood discs, spirodienone linkages ranged from 0.85-1.66% and β-5 linkages ranged from 2.19-3.56% abundance. Additionally, there was generally a neutral or negative correlation between the β-5 and β-β linkages, suggesting there may be a trade-off between the abundance of condensed C-C linkages.

The PB content varied widely among the four genotypes and the region type, though the PB content was generally lower in reaction zone and/or infected region than in healthy region. This reduced PB content in the reaction zone is consistent with observed response to wounding (Frankenstein et al., 2006). In genotype BESC-144, the PB content of the infected region was approximately 50% lower than that of the healthy region. Similarly, BESC-335 showed a reduction in PB content from 6.42% in the healthy region to 1.74% in the infected region. The PB content of genotype 13127 was slightly higher in the infected region (2.96%) than the reaction zone region (2.01%), though both were lower than the healthy region (4.80%). However, HOMC-21-5 exhibited the opposite trend. While HOMC-21-5 measured a PB content of 3.69% in the healthy region and 1.76% in the reaction zone region, the infected region exhibited a PB content of 8.22%. Many aspects regarding the biosynthesis and function of PB remain a mystery. As PB primarily acylates the γ-position of S units, and it has long been hypothesized that PB promotes the formation of S-rich lignin. However, there was no correlation between S and PB abundance (*p*-value = 0.56). A recent study has also called this hypothesis into question (Mottiar and Mansfield, 2022). There is some evidence that PB production is associated with fungal infections (Schnitzler and Seitz, 1989), though that contradicts the trend observed here. Frankenstein et al. reported that wounding lead to decreased *p*-hydroxybenzoic acid content (Frankenstein et al., 2006), which is more consistent with these observations. While there appears to be appreciable PB variability among the twelve samples, based on the current knowledge (or lack thereof) of PB biosynthesis, it is difficult to determine if variation in levels is causative or merely correlative.

### 4.3 Klason lignin analysis

The expected general trend of increased lignification was observed in each of the four infected *Populus* genotypes in this study by analyzing the Klason lignin content of the healthy, reaction zone, and infected region of each genotype. It has been previously shown that *Populus* forms a lignin rich periderm around the site of *Septoria* infection for containment. This is consistent with the trend observed in **Figure 4**, with the reaction zone and/or infected regions generally having a higher Klason lignin content than the healthy region. The most pronounced difference in Klason lignin content was observed in BESC-335, which exhibited the most severe and consistent degree of infection (**Figure 1**). In this sample, both the reaction zone and infected regions exhibited significantly higher lignin content (*p*-value < 0.05) than the healthy region. Similarly, sample BESC-144 exhibited higher lignin content in the infected region. These results signify the recruitment of lignin (or possibly lignin-like phenolics) toward a periderm to contain the fungal infection. Genotype 13127 exhibited a general increase in lignin content from healthy to reaction zone to infected region. However, this increase was determined to not be statistically significant. This is primarily driven by the high variability of the infected region (σ=3.9%) compared to the healthy region (σ=0.9%). Genotype HOMC-21-5 displayed a trend slightly different from the other three lines. The reaction zone had the highest lignin content, while the healthy and infected regions exhibited lower and similar lignin content. Increased lignin content was also an observed response from wounding of *Populus* (Frankenstein et al., 2006). From **Figure 1**, it appears that HOMC-21-5 had the lowest severity of infection. Poplar species exhibit various degrees of susceptibility toward *Septoria* infection. Several factors can influence the severity of *Septoria* infection. For instance, there may be pathogen interaction effects with certain *Populus* genotypes (Ward & Ostry, 2005). Additionally, environmental conditions at the time of infection can also influence severity (Ward & Ostry, 2005). These factors may influence recruitment of lignin to form a periderm to contain an infection, resulting in higher lignin content at the reaction zone between the healthy and infection region. Still, this result contrasts the trends of the other three lines, where the infected regions exhibited notably higher lignin content than the healthy regions. However, these results align with the NMR results, as the reaction zone sample exhibited both the highest S/G ratio (2.83) and the highest lignin content (24.87%) of the HOMC-21-5 line. Likewise, the healthy and infected regions had lower S/G ratios (2.50, 2.38), corresponding to their lower lignin content (19.12%, 21.36%). The area of the infected site could explain this outcome. HOMC-21-5 exhibited a less severe degree of infection which may have impacted the ability to collect the proper region. Additionally, the infection may not have been consistent throughout the thickness of the wood discs. It is therefore possible that healthy region may have been present in the infected material of HOMC-21-5 which would have an impact on the measurement. It is known that *Populus* will develop necrophylactic periderm (NP) layers around the sites of fungal infections to prevent the pathogen’s spread (Qin and LeBoldus, 2014). These NP layers are created by depositing lignin and lignin-like phenolic compounds such as suberin (Mullick, 1977). A study of poplar confirmed upregulation of several lignin biosynthesis genes after being infected with *Sphaerulina*, including CoA 3-O-methyltransferase (CCoAMT), cinnamoyl CoA reductase, (CCR), and cinnamyl alcohol dehydrogenase (CAD) (Foster et al., 2015). As such, the lignin content of infected regions is expected to be higher than in the healthy regions. However, to our knowledge, increased lignification due to *Septoria* infection in poplar has not been analyzed by quantitative methods.

### 4.4 Conclusion

The fungus *Septoria musiva* poses a serious threat to the productivity of economically important poplar. While various studies have explored short-term changes in the days following inoculation, the long-term phenotypic changes of lignin in field-grown, naturally infected poplar stems from *Septoria* canker have not been previously reported. FTIR spectroscopy indicated there were there were likely changes to lignin content and/or structure between healthy and reaction zone/infected region, though trends were not consistent across all samples. Subsequent PCA of whole cell wall biomass samples identified that reaction zone and infected regions are similar, and can be distinguished from healthy samples. Likewise, two samples were found to have significantly higher lignin content in the infected region, and one of these also had significantly higher lignin content in the reaction zone. HSQC NMR examined differences in lignin structure. While differences in lignin structure were observed, there did not appear to be a uniform trend between region types. Two samples with elevated PB content were detected by NMR, which was validated by alkaline hydrolysis and HPLC. Whole cell wall HSQC NMR analysis of one sample suggest that polysaccharide structures remain similar across region types Additionally, the Klason lignin content trended well with the S/G ratio. These results corroborate the observations of previous studies, which noted differentially expressed lignin biosynthesis or increased lignification *via* staining after *Septoria* inoculation. A better understanding of lignin response to fungal infections can lead to improved resilience and higher biomass yield of *Populus*, which is critical for the successful implementation of biofuel production.

## Notice

This manuscript has been authored by UT-Battelle, LLC under Contract No. DE-AC05-00OR22725 with the U.S. Department of Energy. The United States Government retains and the publisher, by accepting the article for publication, acknowledges that the United States Government retains a non-exclusive, paid-up, irrevocable, world-wide license to publish or reproduce the published form of this manuscript, or allow others to do so, for United States Government purposes. The Department of Energy will provide public access to these results of federally sponsored research in accordance with the DOE Public Access Plan (http://energy.gov/downloads/doe-public-access-plan). The views and opinions of the authors expressed herein do not necessarily state or reflect those of the United States Government or any agency thereof. Neither the United States Government nor any agency thereof, nor any of their employees, makes any warranty, expressed or implied, or assumes any legal liability or responsibility for the accuracy, completeness, or usefulness of any information, apparatus, product, or process disclosed, or represents that its use would not infringe privately owned rights.

## Data availability statement

The original contributions presented in the study are included in the article/**Supplementary Material**. Further inquiries can be directed to the corresponding author.

## Author contributions

AR and WM conceptualized the study. WM and J-GC established and maintained the field site. NB and YP prepared and analyzed the samples. RW and JB conducted the lignin analyses by GS-MS. NB drafted the manuscript. All authors contributed to the article and approved the submitted version.

## Author disclaimer

The views and opinions of the authors expressed herein do not necessarily state or reflect those of the United States Government or any agency thereof. Neither the United States Government nor any agency thereof, nor any of their employees, makes any warranty, expressed or implied, or assumes any legal liability or responsibility for the accuracy, completeness, or usefulness of any information, apparatus, product, or process disclosed, or represents that its use would not infringe privately owned rights.
