# Supplementary material for: Cell wall response of field grown Populus to Septoria infection
Source: Front Plant Sci. 2023 Jun 7;14:1089011. doi: 10.3389/fpls.2023.1089011 (PMC10282658; doi:10.3389/fpls.2023.1089011)
Supplement: Supplementary file 1 [file DataSheet_1.docx]

Supplemental information for

Cell Wall Response of Field Grown *Populus* to *Septoria* Infection

**Nathan Bryant^1^, Wellington Muchero^2^, Rachel A. Weber^3^, Jaime Barros^3^, Jin-Gui Chen^2^, Timothy J. Tschaplinski^2^, Yunqiao Pu^2^, Arthur J. Ragauskas^1,2,4*^**

^1^ Department of Chemical and Biomolecular Engineering, University of Tennessee, Knoxville, Tennessee 37996, United States

^2^ BioEnergy Science Center & Center for Bioenergy Innovation, Biosciences Division, University of Tennessee-Oak Ridge National Laboratory Joint Institute for Biological Science, Oak Ridge National Laboratory, Oak Ridge, TN 37831, USA

^3^ Division of Plant Sciences and Interdisciplinary Plant Group, University of Missouri, Columbia, MO 65201, United States.

^4^ Department of Chemical and Biomolecular Engineering, University of Tennessee, Center for Renewable Carbon, Department of Forestry, Wildlife, and Fisheries, University of Tennessee Institute of Agriculture, Knoxville, TN 37996 (USA)

*** Correspondence:**Arthur J. Ragauskas
[aragausk@utk.edu](mailto:aragausk@utk.edu)

Contents

Table S1 – Lignin composition results from thioacidolysis and NMR analysis

Figure S1 – Bivariate plot of S/G ratio measured by NMR and thioacidolysis

Figure S2 – Bivariate plot of H unit content measured by NMR and thioacidolysis

Figure S3 – Comparison of PB measurement by NMR and HPLC

Table S2 – HPLC and HSQC NMR measurements of PB content in lignin

Figure S4 – HSQC NMR spectra including healthy control sample GW-9763

Table S3 - HSQC NMR spectra integration results including healthy control sample GW-9763

Figure S5 – Klason lignin content with healthy control sample GW-9763

Figure S6 – Whole cell wall HSQC NMR spectra of BESC-335

Figure S7 – Score plot of PCA output from FTIR spectra

Table S4 – Assignment of peaks from Figure S6

Table S5 – Average and standard deviation of technical replicates analyzed by alkaline hydrolysis and HPLC

Table S6 – Results from internal protocol for analyzing technical replicates of biomass lignin by thioacidolysis

Table S7 – Summary of analyses performed on each sample

Table S8 – Results from internal protocol for analyzing technical replicates of lignin from a standard *Populus* biomass by HSQC NMR

**Table S1** – Lignin composition results from thioacidolysis and NMR analysis

|  |  | **% (Thioacidolysis)** | | | | **% (NMR)** | | | |
| --- | --- | --- | --- | --- | --- | --- | --- | --- | --- |
| **Genotype** | **Status** | **H** | **G** | **S** | **S/G** | **H** | **G** | **S** | **S/G** |
| BESC-76 | Healthy | 0.42 | 35.29 | 64.29 | 1.82 | 1.23 | 29.63 | 69.15 | 2.33 |
| BESC-76 | Healthy | 0.32 | 34.30 | 65.37 | 1.91 | 1.33 | 27.87 | 70.81 | 2.54 |
| BESC-76 | Healthy | 0.36 | 33.93 | 65.71 | 1.94 | 0.90 | 29.65 | 69.45 | 2.34 |
| 13127 | Healthy | 0.60 | 34.21 | 65.20 | 1.91 | 1.11 | 31.46 | 67.43 | 2.14 |
| 13127 | Reaction Zone | 0.76 | 33.22 | 66.02 | 1.99 | 1.03 | 31.33 | 67.65 | 2.16 |
| 13127 | Infected | 0.64 | 35.27 | 64.08 | 1.82 | 1.95 | 29.87 | 68.18 | 2.28 |
| HOMC-21-5 | Healthy | 0.44 | 33.48 | 66.08 | 1.97 | 1.52 | 28.13 | 70.35 | 2.50 |
| HOMC-21-5 | Reaction Zone | 0.42 | 32.86 | 66.73 | 2.03 | 1.50 | 25.75 | 72.75 | 2.83 |
| HOMC-21-5 | Infected | 0.49 | 33.96 | 65.55 | 1.93 | 1.81 | 29.08 | 69.11 | 2.38 |
| BESC-144 | Healthy | 0.57 | 33.07 | 66.36 | 2.01 | 2.55 | 28.96 | 68.49 | 2.37 |
| BESC-144 | Reaction Zone | 0.66 | 35.00 | 64.33 | 1.84 | 2.36 | 29.95 | 67.69 | 2.26 |
| BESC-144 | Infected | 1.10 | 34.91 | 63.99 | 1.83 | 1.18 | 29.43 | 69.39 | 2.36 |
| BESC-335 | Reaction Zone | 0.51 | 33.26 | 66.22 | 1.99 | 1.35 | 27.56 | 71.09 | 2.58 |
| BESC-335 | Infected | 0.56 | 31.52 | 67.92 | 2.15 | 1.93 | 26.63 | 71.44 | 2.68 |
| GW-9763 | Healthy | 0.89 | 32.61 | 66.50 | 2.04 | 1.36 | 28.10 | 70.54 | 2.51 |
| GW-9763 | Healthy | 0.42 | 32.08 | 67.50 | 2.10 | 1.98 | 26.24 | 71.77 | 2.73 |
| GW-9763 | Healthy | 0.42 | 32.62 | 66.96 | 2.05 | 1.93 | 28.92 | 69.16 | 2.39 |
| *Note: samples BESC-144 Infected and GW-9763 healthy yielded low level of internal standard and lignin* | | | | | | | | | |

**Figure S1** – Bivariate plot of S/G ratio measured by NMR and thioacidolysis

**Figure S2** – Bivariate plot of H unit content measured by NMR and thioacidolysis

**Figure S3** – Comparison of PB measurement by NMR and HPLC

All samples were analyzed by HSQC NMR. It is known that HSQC NMR tends to overestimate PB content. Therefore, samples were subjected to alkaline hydrolysis and HPLC analysis to provide a quantitative measurement of PB content and validate the HSQC NMR results. For the HPLC measurement, PB content is normalized by Klason lignin content. Samples (🌕) exhibited good correlation (R^2^=0.712) between NMR and HPLC measurements. Due to material availability, not all samples were analyzed by HPLC. Additionally, running samples in duplicate was not possible. Therefore, a sample referred to as BESC standard poplar (▲) was utilized for replicate runs to estimate the variability for both HPLC and HSQC NMR. This reference material has been utilized in previous studies and was measured to have a Klason lignin content of 24.5%. The PB content of BESC standard poplar was measured to be 2.13% ± 0.20% by duplicate HPLC analysis, and 16.27% ± 0.36% by triplicate HSQC NMR analysis. As the subset of ten samples from this study were unable to be run in duplicate, the variability from this sample was utilized for error bars. The trendline in Figure S3 was established using only samples (i.e., excluding the BESC standard reference). The BESC standard reference is in excellent agreement with this trendline, providing confidence that this method can validate across a wide range of PB content. If the BESC standard reference is included when generating the trendline, the R^2^ value increases to approximately 0.91.

**Table S2** – HPLC and HSQC NMR measurements of PB content in lignin

|  |  | **PB in lignin** | |
| --- | --- | --- | --- |
| **Genotype** | **Region** | **HPLC** | **HSQC NMR** |
| 13127 | Infected | 0.73% | 2.96% |
| HOMC-21-5 | Healthy | 1.06% | 3.69% |
| HOMC-21-5 | Reaction Zone | 0.76% | 1.76% |
| HOMC-21-5 | Infected | 1.44% | 8.22% |
| BESC-335 | Healthy | 1.22% | 6.42% |
| BESC-335 | Reaction Zone | 0.83% | 3.94% |
| BESC-335 | Infected | 0.80% | 1.74% |
| GW-9763 | Healthy | 0.79% | 1.64% |
| GW-9763 | Healthy | 0.90% | 1.61% |
| GW-9763 | Healthy | 1.16% | 2.83% |

**Figure S4** – HSQC NMR spectra including healthy control sample GW-9763


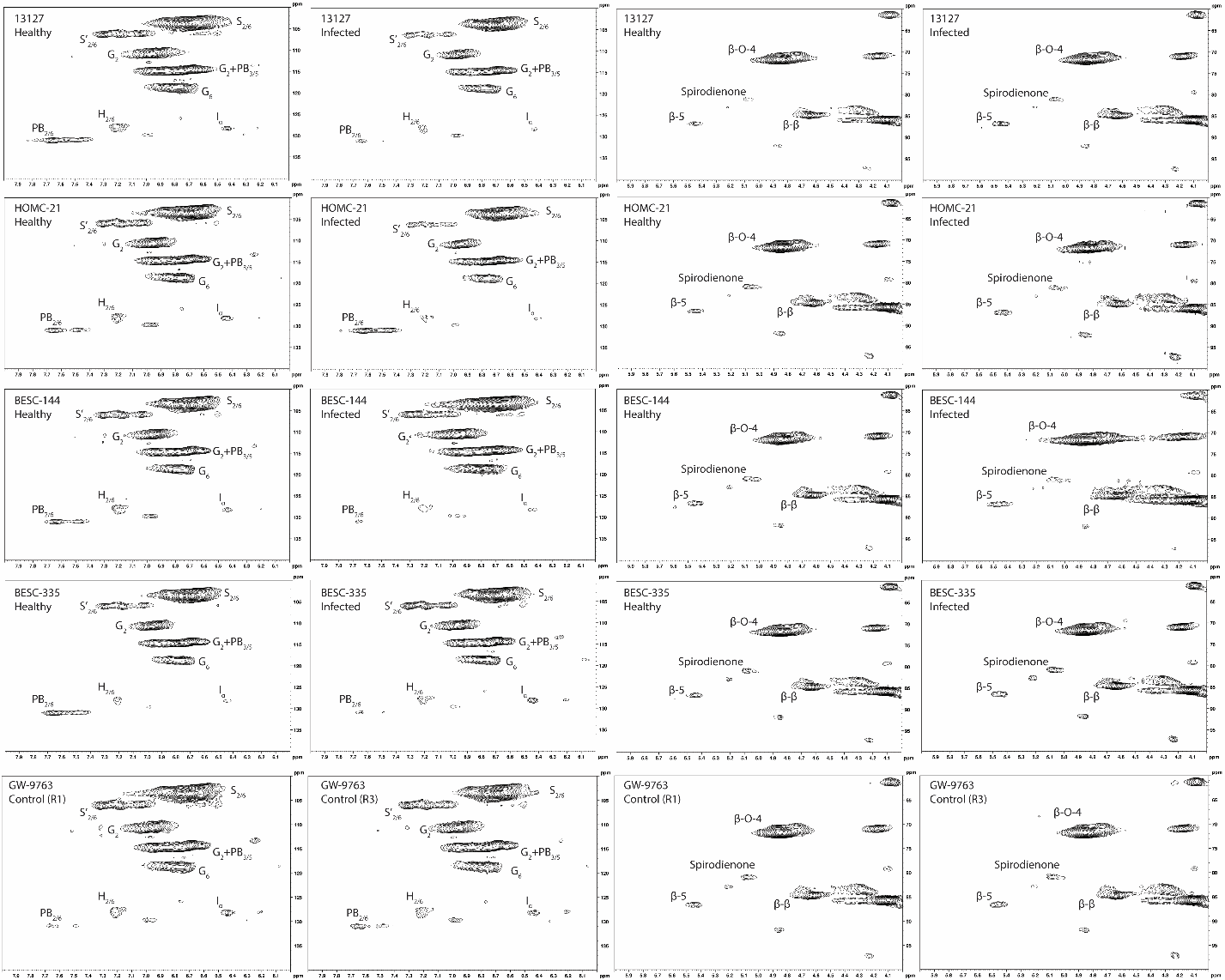


**Table S3** - HSQC NMR spectra integration results including healthy control sample GW-9763

| **Sample** | **Status** | **S** | **G** | **H** | **PB** | **S/G** | **β-O-4** | **β-5** | **β-β** | **Spirodienone** |
| --- | --- | --- | --- | --- | --- | --- | --- | --- | --- | --- |
| 13127 | Healthy | 67.4 | 31.5 | 1.1 | 4.8 | 2.1 | 58.8 | 3.6 | 7.6 | 1.3 |
| 13127 | Reaction Zone | 67.7 | 31.3 | 1.0 | 2.0 | 2.2 | 60.1 | 3.2 | 8.5 | 1.3 |
| 13127 | Infected | 68.2 | 29.9 | 2.0 | 3.0 | 2.3 | 57.9 | 3.2 | 8.1 | 1.4 |
| HOMC-21 | Healthy | 70.4 | 28.1 | 1.5 | 3.7 | 2.5 | 60.4 | 2.7 | 7.5 | 1.6 |
| HOMC-21 | Reaction Zone | 72.8 | 25.8 | 1.5 | 1.8 | 2.8 | 62.2 | 2.2 | 8.4 | 1.0 |
| HOMC-21 | Infected | 69.1 | 29.1 | 1.8 | 8.2 | 2.4 | 57.2 | 2.5 | 5.7 | 0.9 |
| BESC-144 | Healthy | 68.5 | 29.0 | 2.6 | 4.3 | 2.4 | 56.9 | 3.0 | 7.5 | 1.7 |
| BESC-144 | Reaction Zone | 67.7 | 30.0 | 2.4 | 3.6 | 2.3 | 64.7 | 3.2 | 8.1 | 1.6 |
| BESC-144 | Infected | 69.4 | 29.4 | 1.2 | 2.0 | 2.4 | 61.3 | 2.9 | 8.1 | 1.3 |
| BESC-335 | Healthy | 70.5 | 27.6 | 1.8 | 6.4 | 2.6 | 58.5 | 3.0 | 8.2 | 1.6 |
| BESC-335 | Reaction Zone | 71.1 | 27.6 | 1.4 | 3.9 | 2.6 | 57.9 | 2.5 | 7.8 | 1.5 |
| BESC-335 | Infected | 71.4 | 26.6 | 1.9 | 1.7 | 2.7 | 58.0 | 2.8 | 8.5 | 1.6 |
| GW-9763 | Healthy | 70.5 | 28.1 | 1.4 | 1.6 | 2.5 | 58.2 | 3.0 | 8.0 | 1.6 |
| GW-9763 | Healthy | 71.8 | 26.2 | 2.0 | 1.6 | 2.7 | 59.0 | 2.7 | 7.9 | 1.7 |
| GW-9763 | Healthy | 69.2 | 28.9 | 1.9 | 2.8 | 2.4 | 58.1 | 3.2 | 7.5 | 1.5 |

**Figure S5** – Klason lignin content with healthy control sample GW-9763

**Figure S6** – Whole cell wall HSQC NMR spectra of BESC-335

Healthy, Reaction Zone, and infected region of BESC-335 was analyzed by whole cell wall (WCW) HSQC NMR by directly dissolving extractive-free, ball-milled biomass in DMSO-d_6_/HMPA-d_18_ (4:1). Polysaccharide signals in the anomeric and non-anomeric regions are shown. While some minor differences were observed, polysaccharide structures are generally conserved.


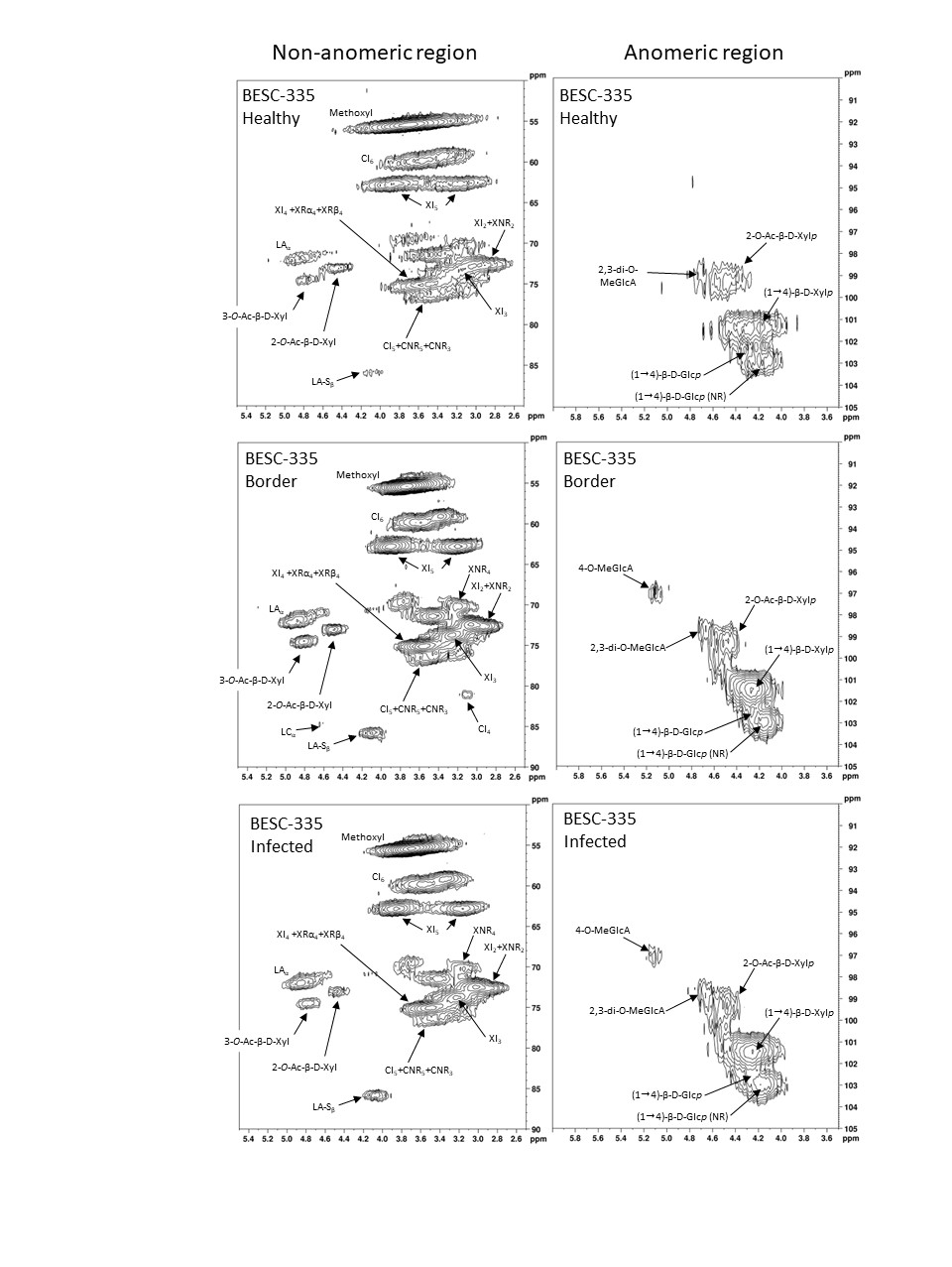


**Figure S7** – Score plot of PCA output from FTIR spectra

Score plot of PC1 and PC2 from the PCA of extracted biomass FTIR spectra. The region from the reaction zone (black) and infected (green) areas are grouped closely together, indicating that these two groups exhibit a high degree of similarity. The associated loading plot corresponds to spectral loading contributions to Principal Component 1 and Principal Component 2. Specific loadings for spectral data available in the supplemental Excel file.


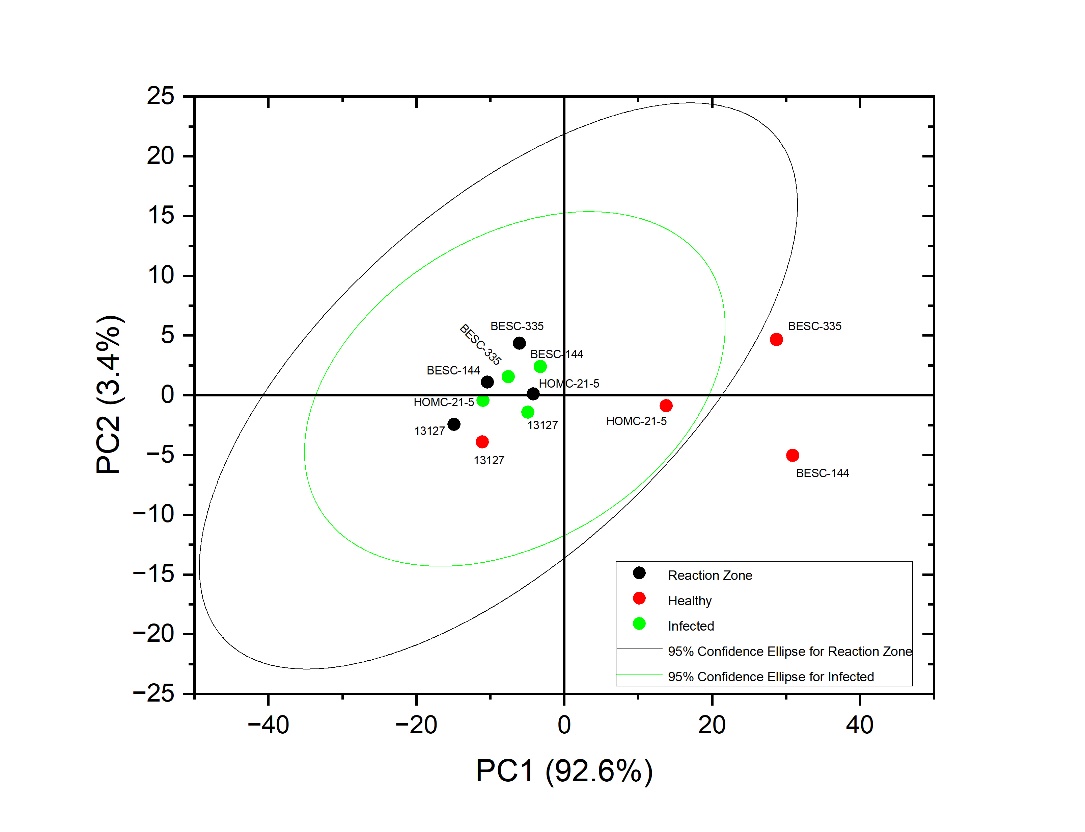


**Table S4** – Assignment of peaks from Figure S6

| **Non-anomeric region** |  |  |  |
| --- | --- | --- | --- |
| **Signal** | **Description** | **δ_H_** | **δ_C_** |
| CI4 | Internal cellulose unit | 3.08 | 81.21 |
| CI5 | Internal cellulose unit | 3.56 | 76.71 |
| CI6 | Internal cellulose unit | 3.56 | 59.95 |
| XI2 | Internal xylose unit | 3.02 | 72.53 |
| XI4 | Internal xylose unit | 3.51 | 75.20 |
| XI5 | Internal xylose unit | 3.17/3.87 | 62.81 |
| XNR_2_ | Xylose non-reducing end | 3.02 | 72.53 |
| XRα_4_ | Xylose reducing end | 3.51 | 75.20 |
| XRβ_4_ | Xylose reducing end | 3.51 | 75.20 |
| CNR_3_ | Cellulose non-reducing end | 3.56 | 76.71 |
| CNR_5_ | Cellulose non-reducing end | 3.56 | 76.71 |
| LA-Sβ | β-O-4 linkage | 4.1 | 85.85 |
| 3-O-Ac-β-D-Xyl | Acetylated xylan | 4.78 | 74.51 |
| 2-*O*-Ac-β-D-Xyl | Acetylated xylan | 4.46 | 73.07 |
| LA_α_ | β-O-4 linkage | 4.85 | 72.11 |
| LC | β-β linkage | 4.65 | 84.73 |
|  |  |  |  |
| **Anomeric region** |  |  |  |
| **Signal** | **Description** | **δ_H_** | **δ_C_** |
| 4-O-MeGlcA | 4-O-methyl-α-ᴅ-glucuronic acid | 5.12 | 96.93 |
| 2,3-di-O-MeGlcA | 2,3-di-O-methyl-a-d-glucuronic acid | 4.71 | 98.75 |
| (1⭢4)-β-D-Glc*p* | Internal cellulose unit | 4.26 | 102.49 |
| (1⭢4)-β-D-Glc*p* (NR) | Cellulose non-reducing end | 4.17 | 102.02 |
| 2-O-Ac-β-D-Xyl*p* | acetylated xylosyl residues at C2 | 4.47 | 99.21 |
| (1⭢4)-β-D-Xyl*p* | Internal xylan unit | 4.26 | 101.53 |

**Table S5** – Average and standard deviation of technical replicates analyzed by alkaline hydrolysis and HPLC

| *BESC Standard Reference Material* | |
| --- | --- |
| PB (HPLC) | 2.13% ± 0.20% (2 replicates) |

| **ID#** | **Sample** | **Rep.** | **H%** | **C%** | **G%** | **S%** | **S/G** |
| --- | --- | --- | --- | --- | --- | --- | --- |
| 1 | Genotype 1 | R1 | 0.82 | 0.82 | 44.40 | 53.96 | 1.22 |
| 2 | Genotype 1 | R2 | 0.70 | 0.86 | 43.77 | 54.67 | 1.25 |
| 3 | Genotype 1 | R3 | 1.08 | 0.74 | 45.79 | 52.39 | 1.14 |
| 4 | Genotype 2 | R1 | 1.20 | 0.66 | 49.07 | 49.06 | 1.00 |
| 5 | Genotype 2 | R2 | 1.30 | 0.66 | 47.95 | 50.10 | 1.04 |
| 6 | Genotype 2 | R3 | 1.48 | 0.63 | 54.02 | 43.87 | 0.81 |
| 7 | Genotype 3 | R1 | 0.86 | 0.20 | 90.66 | 6.18 | 0.07 |
| 8 | Genotype 3 | R2 | 0.49 | 0.15 | 94.87 | 4.48 | 0.05 |
| 9 | Genotype 3 | R3 | 0.52 | 0.15 | 95.15 | 4.18 | 0.04 |
| 10 | Genotype 4 | R1 | 1.39 | 0.43 | 79.56 | 18.61 | 0.23 |
| 11 | Genotype 4 | R2 | 1.08 | 0.30 | 83.50 | 15.11 | 0.18 |
| 12 | Genotype 4 | R3 | 1.01 | 0.37 | 77.36 | 21.25 | 0.27 |

**Table S6** – Results from internal protocol for analyzing technical replicates of biomass lignin by thioacidolysis

| **Genotype** | **Region** | **Enzyme lignin HSQC NMR  (1 replicate)** | **Klason Lignin**  **(2 replicates)** | **FTIR  (3 replicates)** | **Thioacidolysis (1 replicate)** | **HPLC  (1 replicate)** | **Whole cell wall HSQC NMR  (1 replicate)** |
| --- | --- | --- | --- | --- | --- | --- | --- |
| 13127 | Healthy | 🗸 | 🗸 | 🗸 | 🗸 |  | 🗸 |
|  | Reaction Zone | 🗸 | 🗸 | 🗸 | 🗸 |  | 🗸 |
|  | Infected | 🗸 | 🗸 | 🗸 | 🗸 | 🗸 | 🗸 |
| HOMC-21-5 | Healthy | 🗸 | 🗸 | 🗸 | 🗸 | 🗸 |  |
|  | Reaction Zone | 🗸 | 🗸 | 🗸 | 🗸 | 🗸 |  |
|  | Infected | 🗸 | 🗸 | 🗸 | 🗸 | 🗸 |  |
| BESC-144 | Healthy | 🗸 | 🗸 | 🗸 | 🗸 |  |  |
|  | Reaction Zone | 🗸 | 🗸 | 🗸 | 🗸 |  |  |
|  | Infected | 🗸 | 🗸 | 🗸 | 🗸 |  |  |
| BESC-335 | Healthy | 🗸 | 🗸 | 🗸 |  | 🗸 |  |
|  | Reaction Zone | 🗸 | 🗸 | 🗸 | 🗸 | 🗸 |  |
|  | Infected | 🗸 | 🗸 | 🗸 | 🗸 | 🗸 |  |
| GW-9763 | Healthy | 🗸 | 🗸 | 🗸 | 🗸 | 🗸 |  |
|  | Healthy | 🗸 | 🗸 | 🗸 | 🗸 | 🗸 |  |
|  | Healthy | 🗸 | 🗸 | 🗸 | 🗸 | 🗸 |  |
| BESC-76 | Healthy | 🗸 | 🗸 | 🗸 | 🗸 |  |  |
|  | Healthy | 🗸 | 🗸 | 🗸 | 🗸 |  |  |
|  | Healthy | 🗸 | 🗸 | 🗸 | 🗸 |  |  |

**Table S7** – Summary of analyses performed on each sample

**Table S8** – Results from internal protocol for analyzing technical replicates of lignin from a standard *Populus* biomass by HSQC NMR

|  | **Mean** | **StDev** |
| --- | --- | --- |
| S | 59.0 | 0.5 |
| G | 40.7 | 0.46 |
| H | 0.33 | 0.2 |
| PB | 16.5 | 0.4 |
| S/G | 1.45 | 0.03 |
| β-O-4 | 61.6 | 0.9 |
| β-5 | 4.01 | 0.12 |
| β-β | 3.93 | 0.74 |
